# Case Report: Pelvic–perineal deep angiomyxoma presenting as hematoma-like paravaginal masses: a report of two cases

**DOI:** 10.3389/fonc.2026.1940480

**Published:** 2026-10-05

**Authors:** Xiaodan Yang, Fang Wang

**Affiliations:** Department of Gynecology of Integrated Traditional Chinese and Western Medicine, Gansu Provincial Maternity and Child-Care Hospital (Gansu Provincial Central Hospital), Lanzhou, Gansu, China

**Keywords:** case report, deep angiomyxoma (DAM), hematoma-like paravaginal masses, magnetic resonance imaging (MRI), misdiagnosis

## Abstract

Deep angiomyxoma (DAM) is a rare, locally infiltrative mesenchymal tumor of the female pelvic and perineal regions that is often misdiagnosed because of nonspecific clinical and ultrasonographic findings. We report two cases of pelvic–perineal DAM initially interpreted as paravaginal hematomas. One patient underwent ultrasound-guided aspiration and thrombin injection before progressive enlargement prompted further evaluation. Magnetic resonance imaging (MRI) demonstrated marked T2 hyperintensity, internal swirling structures, and extension along pelvic floor tissue planes, raising suspicion of DAM. Both patients underwent surgical resection with histologically negative margins (R0). Histopathology confirmed DAM with estrogen receptor and progesterone receptor positivity. Following multidisciplinary discussion, both patients received six cycles of individualized postoperative gonadotropin-releasing hormone agonist therapy. No radiological recurrence was observed at 11 and 12 months, respectively. Persistent or enlarging hematoma-like pelvic–perineal masses without a clear precipitating factor should prompt consideration of DAM and further MRI evaluation. MRI is useful for suggesting DAM and delineating tumor extent, whereas definitive diagnosis relies on histopathology. The benefit of routine postoperative endocrine therapy after R0 resection remains uncertain.

## Introduction

Deep angiomyxoma (DAM), historically termed aggressive angiomyxoma (AAM), is a rare mesenchymal soft-tissue tumor first described by Steeper and Rosai in 1983 (1, 2). Histologically, it is characterized by sparsely distributed spindle-shaped or stellate cells embedded in an abundant myxoid stroma containing blood vessels of variable caliber (2). DAM predominantly affects women of reproductive age and commonly involves the pelvis, perineum, perianal region, and vagina. Although it generally grows slowly, it exhibits marked local infiltrative behavior, with reported postoperative recurrence rates of 36%–72%, whereas distant metastasis is rare (3, 4). Because of its nonspecific clinical manifestations, DAM may be misdiagnosed as a Bartholin gland cyst, hematoma, lipoma, leiomyoma, or inguinal hernia.

Surgical resection remains the mainstay of treatment (5), although complete excision can be challenging because of deep pelvic location, ill-defined margins, and proximity to critical structures. Preoperative MRI, multidisciplinary evaluation, and individualized surgical planning are therefore important. Hormonal therapy may be considered in selected patients with unresectable, residual, or recurrent hormone receptor-positive disease. Here, we report two cases of DAM presenting as hematoma-like paravaginal masses, highlighting the diagnostic consequences of repeated ultrasonographic misinterpretation, including aspiration and thrombin injection in Case 1, as well as the value of longitudinal imaging assessment and multidisciplinary perioperative management.

## Case report

### Case 1

A 37-year-old woman (gravida 2, para 2) was admitted on May 30, 2025, with a 2-year history of perineal swelling and discomfort and a 2-month history of pain. Her menstrual cycles were regular, although the menstrual flow was relatively scant. Two years before admission, she had developed intermittent perineal swelling without an identifiable precipitating factor. She initially sought medical attention at another hospital, where a Bartholin gland cyst was suspected. In June 2023, transvaginal ultrasonography revealed a mixed-echoic mass measuring approximately 70 × 65 × 50 mm on the left side of the vagina, which was interpreted as a hematoma (**Figure 1A**). Based on this presumed diagnosis, ultrasound-guided aspiration was performed, followed by thrombin injection. Follow-up ultrasonography in August 2023 showed a reduction in the size of the lesion to approximately 67 × 52 × 42 mm, and a thrombosed venous pseudoaneurysm was subsequently suspected (**Figure 1B**). The patient did not undergo regular follow-up thereafter.

**Figure 1:**
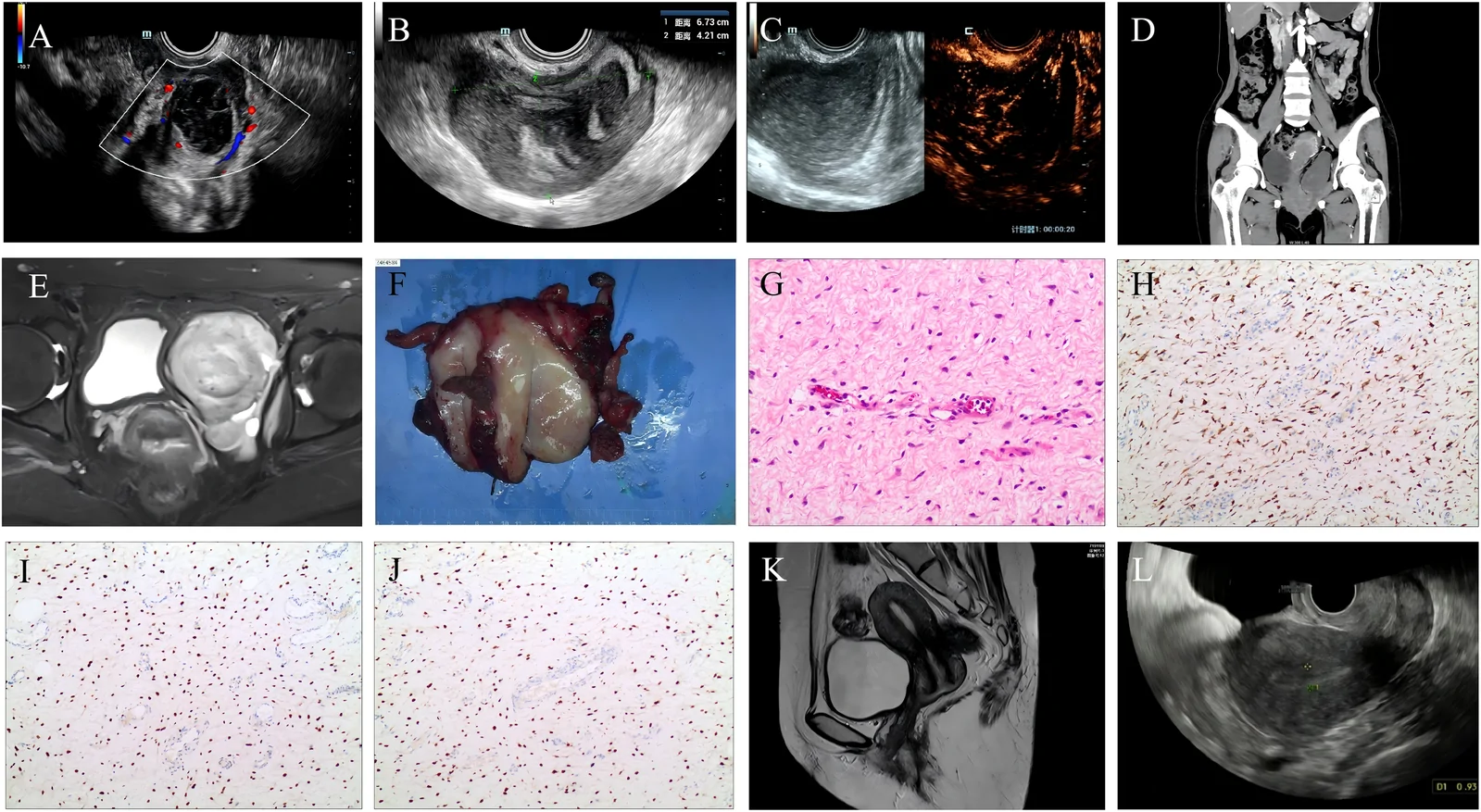
Diagnostic, surgical, histopathological, and follow-up findings in Case 1. **(A)** Transvaginal ultrasonography in June 2023 showed a mixed-echoic left paravaginal mass, initially interpreted as a hematoma-like paravaginal mass. **(B)** Follow-up ultrasonography in August 2023 showed a smaller lesion, with a thrombosed venous pseudoaneurysm subsequently suspected. **(C)** Contrast-enhanced ultrasonography in May 2025 showed marked lesion enlargement with heterogeneous enhancement. **(D)** Contrast-enhanced CT demonstrated an irregular hypodense left pelvic–paravaginal mass. **(E)** Pelvic T2-weighted MRI showed a markedly hyperintense mass with internal strand-like and swirling hypointense structures. **(F)** Gross surgical specimen. **(G)** Hematoxylin and eosin staining showed sparsely distributed spindle-shaped and stellate tumor cells embedded in a vascular myxoid stroma (×200). **(H–J)** Immunohistochemical staining demonstrated cytoplasmic positivity for desmin and nuclear positivity for estrogen receptor and progesterone receptor (×200). **(K)** Pelvic MRI on January 29, 2026, showed no definite residual or recurrent lesion. **(L)** Transvaginal ultrasonography on April 30, 2026, showed no abnormal mass.

In March 2025, the patient developed perineal pain and discomfort. Contrast-enhanced ultrasonography performed at our outpatient clinic in May 2025 demonstrated marked enlargement of the mixed-echoic mass on the left side of the vagina to approximately 107 × 100 × 62 mm, with internal layered echoes, raising suspicion of DAM (**Figure 1C**). She was therefore admitted for further evaluation and treatment. Gynecological examination revealed prominent swelling of the left buttock near the perineum, with no other significant abnormalities. Contrast-enhanced computed tomography of the abdomen demonstrated an irregular hypodense mass involving the left pelvic sidewall and left vaginal region (**Figure 1D**). Pelvic magnetic resonance imaging (MRI) revealed an irregular mass extending from the left vaginal wall into the left pelvic cavity, measuring approximately 114 × 90 × 67 mm. The lesion was markedly hyperintense on T2-weighted images and showed heterogeneous enhancement after contrast administration, with imaging features suggestive of DAM (**Figure 1E**). Compared with the imaging findings obtained in 2023, the lesion had increased substantially in size. Routine biochemical tests, serum tumor markers, human papillomavirus testing, and liquid-based cervical cytology showed no significant abnormalities.

Following multidisciplinary consultation, the lesion was considered highly suspicious for DAM and amenable to surgical resection based on its imaging features and anatomical extent. Preoperative tissue biopsy was not performed. Given the deep paravaginal and pelvic extension of the tumor and its close relationship with the left ureter, a prophylactic left ureteral stent was placed preoperatively. On June 4, 2025, the patient underwent laparoscopic-assisted transvaginal tumor resection. Intraoperatively, the tumor extended superiorly from the left paravaginal space toward the broad ligament and was highly vascular. Macroscopically complete resection was achieved. The resected pelvic–paravaginal specimen measured 14.5 × 8 × 4 cm on gross examination (**Figure 1F**). Histopathological examination confirmed DAM with negative resection margins (R0 resection) (**Figure 1G**). Microscopically, the tumor was a hypocellular myxoid neoplasm composed of bland spindle-shaped to stellate stromal cells embedded in abundant myxoid stroma, with scattered vascular channels. No significant cytological atypia or conspicuous mitotic activity was identified. Immunohistochemical staining showed positivity for desmin, smooth muscle actin, estrogen receptor (ER), and progesterone receptor (PR), with a Ki-67 proliferation index of <5% (**Figures 1H–J**).

On postoperative day 6, the patient developed mild vaginal bleeding accompanied by pain. Examination revealed partial dehiscence of the left vaginal wall incision, and bacterial culture of the wound discharge grew Escherichia coli. The incision gradually healed following debridement, dressing changes, and culture-directed antimicrobial therapy.

Given the patient’s premenopausal status, the large and deeply located tumor with locally infiltrative behavior, and ER/PR positivity, postoperative gonadotropin-releasing hormone agonist (GnRHa) therapy was selected after multidisciplinary discussion as an individualized treatment strategy. The patient received six cycles of leuprolide acetate microspheres (3.75 mg subcutaneously every 28 days). Because of heightened concern regarding recurrence, the patient underwent more frequent follow-up and was reviewed approximately monthly, resulting in 12 follow-up visits during the first postoperative year. Follow-up imaging at our institution included transabdominal gynecologic ultrasonography on July 24, 2025, transvaginal ultrasonography on October 29, 2025, pelvic MRI on January 29, 2026, and transvaginal ultrasonography on April 30, 2026. The pelvic MRI obtained on January 29, 2026, and transvaginal ultrasonography obtained on April 30, 2026, are shown in **Figures 1K, L**, respectively. At the latest follow-up on April 30, 2026, the patient was in good general condition, with no radiological evidence of tumor recurrence. The clinical timeline is presented in **Figure 2**.

**Figure 2:**
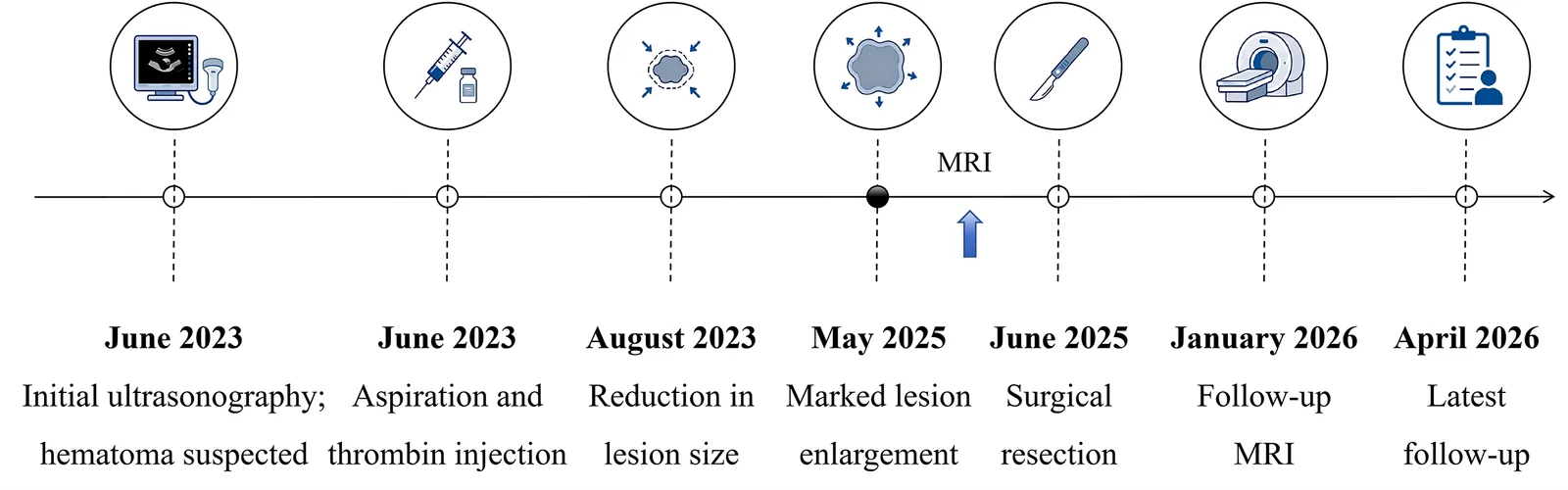
Clinical timeline of Case 1.

### Case 2

A 46-year-old woman (gravida 4, para 3) was admitted on April 8, 2025, with a 1-month history of a left vulvar mass accompanied by a sensation of incomplete bladder emptying. Her menstrual cycles were regular. Ultrasonography performed at a local hospital revealed a large hypoechoic mass measuring approximately 96 × 84 × 74 mm in the left anterior vaginal wall, and she was referred to our hospital for further evaluation.

Gynecological examination revealed an approximately 7-cm cystic mass along the left vaginal wall extending toward the left pelvic cavity and buttock, with mild bulging of the left buttock. Ultrasonography demonstrated a hypoechoic lesion between the left vaginal wall and the levator ani muscle, which was initially interpreted as a hematoma (**Figure 3A**). Pelvic MRI revealed an approximately 10-cm mass in the left pelvic cavity extending along the levator ani space. The lesion showed characteristic layered hyperintensity on T2-weighted images, raising suspicion of DAM (**Figures 3B, C**). A FIGO type 3 uterine leiomyoma and small bilateral ovarian cysts were also identified. Contrast-enhanced abdominal computed tomography demonstrated a left pelvic mass. In conjunction with the MRI findings, a mesenchymal tumor, particularly DAM, was considered (**Figure 3D**). Serum tumor marker levels were within normal limits.

**Figure 3:**
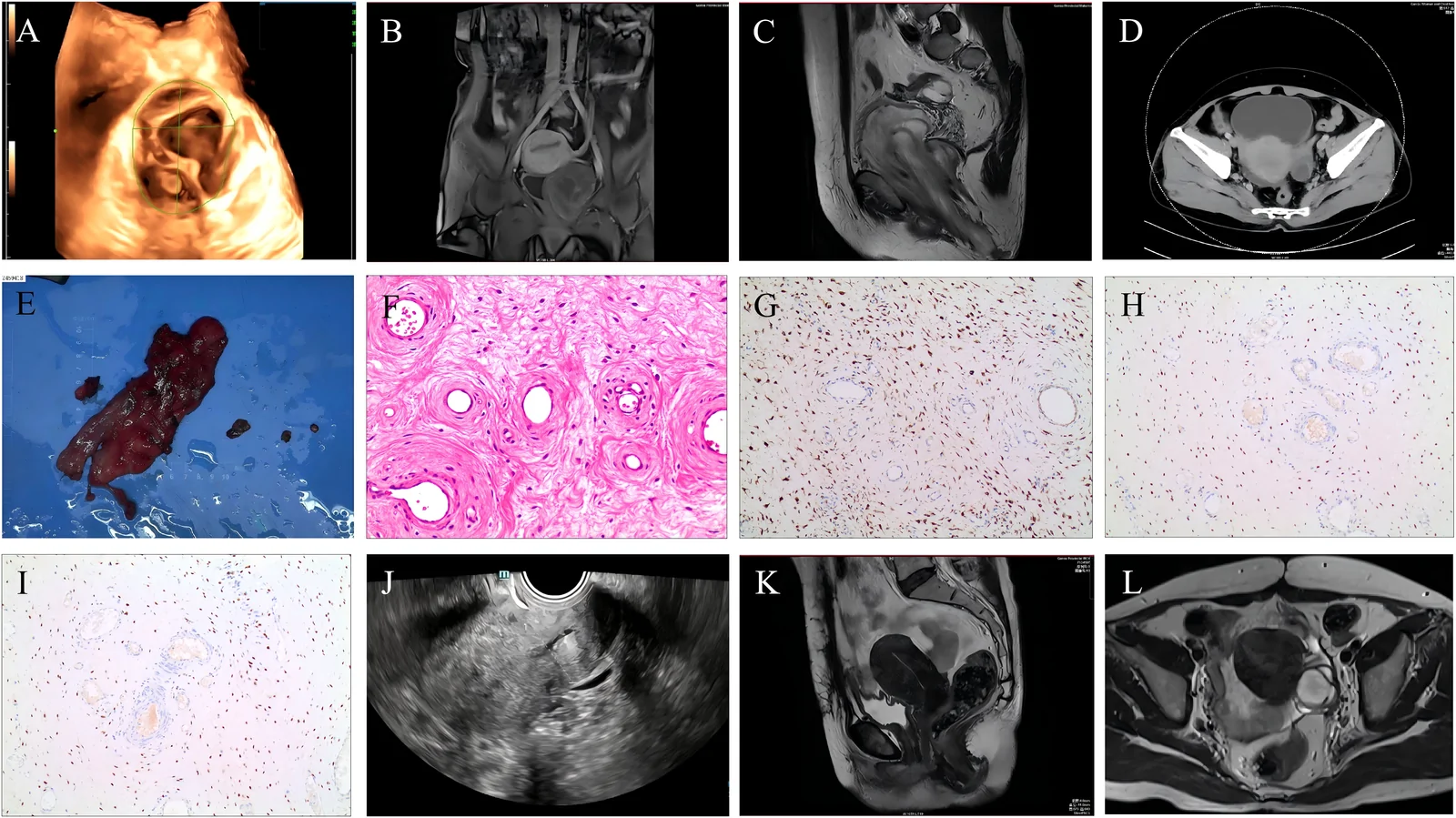
Diagnostic, surgical, histopathological, and follow-up findings in Case 2. **(A)** Three-dimensional transvaginal ultrasonography showed a mixed solid–cystic echogenic mass in the left paravaginal region, initially interpreted as a paravaginal hematoma. **(B, C)** Coronal **(B)** and sagittal **(C)** pelvic T2-weighted MRI demonstrated a lobulated mass in the left pelvic cavity extending along the pelvic floor tissue planes. The lesion was predominantly hyperintense, with internal layered, strand-like, and swirling hypointense structures, suggestive of DAM. **(D)** Pelvic CT showed an irregular soft-tissue mass posterior to the uterus extending into the left pelvic cavity. **(E)** Gross appearance of the resected surgical specimen. **(F)** Hematoxylin and eosin staining showed sparsely distributed spindle-shaped and stellate tumor cells embedded within a vascular myxoid stroma (×200). **(G–I)** Immunohistochemical staining demonstrated cytoplasmic positivity for desmin and nuclear positivity for estrogen receptor and progesterone receptor (×200). **(J)** Transvaginal ultrasonography on December 11, 2025, showed no definite evidence of recurrence. **(K, L)** Pelvic MRI on April 13, 2026, showed no definite residual or recurrent lesion.

Following multidisciplinary consultation, a combined transabdominal and transvaginal approach was selected because the tumor extended from the left vaginal wall along the pelvic floor toward the ischiorectal fossa and buttock, allowing adequate exposure and complete resection while protecting adjacent structures. Preoperative tissue biopsy was also not performed. On April 15, 2025, the patient underwent combined transabdominal and transvaginal tumor resection, myomectomy, left ovarian cystectomy, and left ureteral stent placement. Macroscopically complete resection was achieved. During resection, the tumor was divided into two portions and submitted separately for pathological examination. The two specimens measured 17 × 5 × 1 cm and 13 × 5.5 × 2 cm, respectively, representing two portions of the same tumor rather than separate lesions (**Figure 3E**). Intraoperative frozen-section examination indicated a mesenchymal tumor.

Postoperative histopathological examination confirmed DAM with negative resection margins (R0 resection) (**Figure 3F**), demonstrating characteristic hypocellular myxoid stroma with bland spindle-to-stellate cells and numerous variably sized blood vessels. Immunohistochemical staining showed positivity for desmin, ER, and PR, focal scattered positivity for smooth muscle actin, and negativity for CD34 and S100. The Ki-67 proliferation index was <5% (**Figures 3G–I**).

On postoperative day 6, the patient developed full-thickness dehiscence of the abdominal incision, together with an *Enterococcus faecalis* infection of the vaginal incision. Culture-directed antimicrobial therapy with piperacillin–tazobactam was administered, and repeated debridement and drainage were performed. Secondary closure of the abdominal incision was undertaken on May 6, 2025, after which the wound healed satisfactorily.

Postoperative GnRHa therapy was also selected after multidisciplinary discussion as an individualized treatment strategy, and the patient received six cycles of leuprolide acetate (3.75 mg subcutaneously every 28 days). The left ureteral stent was removed without complication on July 18, 2025, 3 months after surgery. The patient underwent six follow-up visits during the first postoperative year according to the planned schedule. Follow-up imaging at our institution included transvaginal ultrasonography on December 11, 2025, and pelvic MRI on April 13, 2026, as shown in **Figures 3J, K, L**, respectively. At the latest follow-up on April 14, 2026, the patient was in good general condition, with no radiological evidence of tumor recurrence. The clinical characteristics, treatment, and follow-up findings of both patients are summarized in **Table 1**.

**Table 1:** Clinical characteristics, diagnosis, treatment, and follow-up of the two patients with DAM.

Clinical and pathological characteristics	Case 1	Case 2
Age, years	37	46
Gravidity and parity	G2P2	G4P3
Chief complaint	Perineal swelling and discomfort for 2 years, with pain for 2 months	Vulvar mass for 1 month
Serum tumor markers	Normal	Normal
Tumor location	Left vaginal wall and left paravaginal space	Left vaginal wall
Previous misdiagnosis	Paravaginal hematoma; thrombosed venous pseudoaneurysm	Paravaginal hematoma
Extent of tumor involvement	Extended from the left paravaginal space into the left pelvic cavity and broad ligament region	Extended along the pelvic floor and levator ani spaces into the ischiorectal fossa and buttock
Maximum tumor diameter, mm	114	100
Main MRI findings	An irregular mass measuring approximately 114 × 90 × 67 mm extending from the left vaginal wall into the left pelvic cavity; markedly hyperintense on T2-weighted images with heterogeneous contrast enhancement	An approximately 10-cm mass in the left pelvic cavity extending along the levator ani space; characteristic layered hyperintensity on T2-weighted images
Preoperative diagnosis	DAM	DAM
Surgical approach	Laparoscopic-assisted transvaginal approach	Combined transabdominal and transvaginal approach
Surgical procedure	Prophylactic left ureteral stent placement followed by laparoscopic-assisted transvaginal tumor resection	Combined transabdominal and transvaginal tumor resection, myomectomy, left ovarian cystectomy, and left ureteral stent placement
Main intraoperative findings	The tumor extended superiorly from the left paravaginal space toward the broad ligament, was highly vascular, and was closely associated with the left ureter	The tumor arose from the left vaginal wall and extended along the pelvic floor tissue planes into the ischiorectal fossa and buttock
Surgical margin	R0, histologically negative	R0, histologically negative
Immunohistochemical findings	Desmin (+), SMA (+), ER (+), PR (+), and Ki-67 proliferation index <5%	Desmin (+), ER (+), PR (+), focal scattered SMA positivity, CD34 (−), S100 (−), and Ki-67 proliferation index <5%
Postoperative complications	Partial dehiscence of the left vaginal wall incision with *Escherichia coli* infection	Full-thickness dehiscence of the abdominal incision and *Enterococcus faecalis* infection of the vaginal incision
Length of hospital stay, days	14	36
Postoperative adjuvant therapy	Six cycles of GnRH agonist therapy	Six cycles of GnRH agonist therapy
Follow-up duration, months	11	12
Follow-up imaging at our institution	Jul 24, 2025: transabdominal gynecologic US; Oct 29, 2025: transvaginal US; Jan 29, 2026: pelvic MRI; Apr 30, 2026: transvaginal US	Dec 11, 2025: transvaginal US; Apr 13, 2026: pelvic MRI
Outcome	No recurrence	No recurrence

Only follow-up imaging examinations performed at our institution and documented in the available medical records are listed.

DAM, deep angiomyxoma; ER, estrogen receptor; PR, progesterone receptor; SMA, smooth muscle actin; T2WI, T2-weighted imaging; GnRH, gonadotropin-releasing hormone; US, ultrasonography.

## Discussion

Deep angiomyxoma (DAM) is a rare mesenchymal soft-tissue tumor of the pelvic and perineal regions that remains prone to delayed diagnosis and misdiagnosis because of its low incidence and nonspecific clinical manifestations. In the present report, both patients initially presented with vaginal or perineal masses that were interpreted as paravaginal hematomas on early imaging. In Case 1, ultrasound-guided aspiration and thrombin injection were performed before progressive enlargement and subsequent MRI findings raised suspicion of DAM.

The primary reason for the frequent misdiagnosis of DAM is the lack of specific clinical manifestations. The tumor predominantly affects women of reproductive age and commonly arises in the vagina, vulva, pelvic floor, or perineum. It usually grows slowly and often presents as localized swelling, a sensation of pelvic or perineal pressure, or a painless mass, thereby resembling common benign conditions such as Bartholin gland cysts, vaginal cysts, or pelvic hernias (5, 6). In the present cases, both patients had soft masses in the paravaginal or vulvar region, and initial ultrasonography demonstrated mixed-echoic or hypoechoic lesions with fluid-containing areas, which contributed to their interpretation as hematomas. Unlike a true hematoma, however, DAM typically shows persistent, gradual enlargement and may extend infiltratively along pelvic floor fascial planes (7). In Case 1, the lesion increased from approximately 7 cm to more than 10 cm over nearly 2 years, whereas Case 2 already had an approximately 10-cm mass at the initial presentation. Such progressive enlargement should prompt clinicians to reconsider the diagnosis and investigate the possibility of DAM.

Second, overlap between the imaging features of DAM and those of certain benign lesions is another important cause of misdiagnosis. Ultrasonography is commonly used as the first-line imaging modality for pelvic and perineal masses because of its accessibility and convenience; however, it has limited ability to characterize the histologic nature of deep soft-tissue tumors. Because DAM contains abundant myxoid stroma and vascular structures of variable caliber, it may appear hypoechoic or heterogeneous on ultrasonography. In addition, intratumoral hemorrhage, myxoid components, and fibrous septa may produce heterogeneous echogenicity resembling that of a hematoma. Therefore, ultrasonography alone may be insufficient to reliably distinguish DAM from a hematoma. MRI should be performed for hematoma-like lesions that lack a clear history of trauma and persist or progressively enlarge.

MRI is the preferred imaging modality for characterizing suspected DAM and assessing its anatomical extent. Typical DAM appears isointense or hypointense on T1-weighted images and markedly hyperintense on T2-weighted images because of its water-rich myxoid stroma. Internal layered or swirling hypointense strands are frequently observed, and contrast-enhanced imaging may demonstrate prominent layered or swirling enhancement, producing the characteristic “swirl sign” (6, 8). This sign reflects the interlacing distribution of collagen fibers and fibrovascular bundles within the myxoid matrix. In addition, DAM characteristically extends along fascial planes and pelvic floor anatomic spaces in an insinuating or infiltrative manner rather than simply compressing adjacent structures (7). In both of our patients, MRI demonstrated extension of the lesions along the paravaginal space, levator ani space, and pelvic floor tissues, together with characteristic swirling internal structures, which raised strong suspicion of DAM. Accordingly, MRI should be strongly considered in the evaluation of unexplained pelvic or perineal soft-tissue masses, particularly in women of reproductive age.

From an imaging perspective, DAM should primarily be distinguished from paravaginal hematoma, benign cystic lesions, leiomyoma, lipoma, and other soft-tissue tumors. Unlike hematomas, which usually have an identifiable precipitating factor and tend to resolve over time, DAM typically demonstrates progressive enlargement and infiltrative extension. Benign cystic lesions are generally well circumscribed and lack the characteristic internal swirling architecture, whereas leiomyomas and lipomas exhibit their respective smooth-muscle and fat signal characteristics on MRI (9).

Recent reports have also highlighted the variable presentation of DAM. Zhang et al. described three atypical cases mimicking benign vulvovaginal lesions, underscoring the limited specificity of clinical and imaging findings alone (10). Histopathological examination remains essential for the definitive diagnosis of DAM. Classic DAM typically presents as a poorly circumscribed myxoid neoplasm composed of bland spindle-to-ovoid stromal cells and numerous variably sized thin- and thick-walled blood vessels, without significant cytological atypia (2, 10). Preoperative tissue biopsy may be considered when the diagnosis remains uncertain or when histological confirmation would alter management. However, limited sampling may be nondiagnostic in DAM because of its hypocellular myxoid morphology. Therefore, the decision to perform preoperative biopsy should be individualized according to the clinical and imaging context.

Immunohistochemically, tumor cells commonly express desmin, ER, and PR, whereas SMA and CD34 expression may be variable and S100 is typically negative (10). Accordingly, immunohistochemical findings should be interpreted in conjunction with the characteristic morphology and infiltrative growth pattern rather than used in isolation. In diagnostically challenging cases, HMGA2 rearrangement or HMGA2 immunohistochemical expression may provide additional supportive evidence, although these findings are not present in all cases of DAM and are not entirely specific (10, 11).

The principal pathological differential diagnoses include angiomyofibroblastoma (AMFB), cellular angiofibroma (CAF), and superficial angiomyxoma. AMFB is generally well circumscribed and more cellular, often showing characteristic perivascular condensation of stromal cells, whereas DAM is typically larger, more deeply located, hypocellular, and infiltrative (12). CAF is also usually well circumscribed and more cellular, with prominent hyalinized vessels, and frequently shows loss of RB1 expression (12). Superficial angiomyxoma, in contrast, arises predominantly in the dermis or superficial subcutaneous tissue and commonly displays a lobulated architecture, abundant myxoid stroma, numerous small thin-walled vessels, and a variable inflammatory infiltrate, often including neutrophils (13). Accordingly, the diagnosis of DAM should integrate tumor location, growth pattern, histomorphology, and immunophenotype, with molecular testing reserved for selected challenging cases.

Complete surgical excision remains the primary treatment modality for DAM (14). However, achieving complete resection can be challenging because DAM frequently arises in the deep pelvic floor, exhibits infiltrative growth with ill-defined margins, and may involve critical adjacent structures, including the ureters, bladder, rectum, pelvic vessels, and nerves. Therefore, careful preoperative assessment and individualized surgical planning are essential to balance oncological control with preservation of adjacent organs and function. Recent reports have similarly emphasized the importance of individualized surgical decision-making in DAM. Aquino et al. reported a pregnancy-associated DAM case and reviewed 24 similar cases from the literature, highlighting the diagnostic challenges, infiltrative behavior, and importance of individualized surgical decision-making (15). In the present study, both patients underwent individualized surgical approaches based on preoperative MRI assessment. Prophylactic ureteral stent placement was performed before or during surgery to facilitate ureteral identification and reduce the risk of ureteral injury. Despite the complexity of tumor location and extent, histologically margin-negative (R0) resection was achieved in both cases.

Postoperative wound morbidity was notable in both patients. Case 1 developed partial vaginal wound dehiscence with surgical site infection, whereas Case 2 developed full-thickness abdominal wound dehiscence together with vaginal wound infection, requiring repeated debridement, drainage, and secondary closure. These complications may have been related to the large tumor size, extensive dissection within the deep pelvic floor, combined surgical approaches, and the creation of substantial postoperative dead space. Although these findings do not establish wound complications as a specific feature of DAM surgery, they highlight the importance of meticulous dead-space management, adequate drainage, tension-free wound closure, and close postoperative infection surveillance in patients undergoing extensive pelvic–perineal resection.

Nevertheless, local recurrence remains an important concern even after complete surgery. In a multicenter international retrospective study (16), 33 patients (92%) underwent complete resection (R0/R1), with a local relapse rate of 50% and a median relapse-free survival of 39 months. Accordingly, long-term clinical and imaging follow-up is warranted. In our institutional practice, follow-up is generally scheduled every 3 months during the first postoperative year, every 6 months during postoperative years 2–5, and annually thereafter, although the actual surveillance schedule may vary according to individual clinical circumstances. No standardized surveillance schedule has been established for DAM, and the imaging modality, interval, and duration of follow-up should be individualized according to patient- and tumor-related factors.

The frequent expression of ER and PR in DAM suggests potential hormone responsiveness. Among the available endocrine approaches, GnRHa therapy was selected because both patients were premenopausal and had ER/PR-positive tumors, making ovarian suppression a biologically reasonable strategy. Aromatase inhibitors and selective ER modulators have also been reported in DAM (17); however, comparative evidence is limited, and no established guideline defines a preferred postoperative endocrine regimen after R0 resection. The six-month course used in our patients was an empirical individualized regimen rather than a guideline-based treatment duration. Therefore, the choice of GnRHa in our patients reflected an individualized multidisciplinary decision rather than a standard treatment recommendation. Given the R0 resections, small sample size, absence of an untreated comparator, and short follow-up period, the absence of recurrence in our patients cannot be attributed to GnRHa therapy. Hormonal therapy may be considered in selected patients, particularly those with unresectable, residual, or recurrent hormone receptor-positive disease (14).

The principal clinical value of this case series lies in highlighting DAM as a potentially misleading hematoma-like paravaginal mass. Persistent or enlarging hematoma-like pelvic–perineal masses without a clear precipitating factor should prompt reconsideration of the diagnosis and further evaluation with MRI. Recognition of this diagnostic pattern may reduce diagnostic delay and help avoid unnecessary intervention.

This report has several limitations. First, only two patients were included, precluding any meaningful evaluation of the comparative effectiveness of different surgical strategies or postoperative endocrine therapy. Second, the follow-up duration was limited to approximately 11–12 months, which is insufficient to assess the long-term risk of recurrence in DAM. Accordingly, the absence of recurrence observed in the present cases should be interpreted only as a short-term follow-up finding. In addition, HMGA2 immunohistochemistry and molecular testing were not performed in either case. Therefore, the principal value of this report lies in highlighting diagnostic pitfalls and perioperative management considerations rather than establishing definitive treatment recommendations.

## Conclusion

Deep angiomyxoma is a rare pelvic–perineal soft-tissue tumor with locally infiltrative growth and a substantial risk of recurrence. DAM should be considered when a hematoma-like pelvic or perineal mass persists or progressively enlarges without a clear history of trauma, childbirth, or surgery. Marked T2 hyperintensity, internal swirling structures, and extension along pelvic floor tissue planes are important MRI clues, although definitive diagnosis requires histopathological confirmation. MRI-based preoperative assessment and individualized surgical planning may facilitate complete resection while preserving adjacent structures. Given the risk of late local recurrence, long-term individualized clinical and imaging follow-up is warranted. However, the benefit of routine adjuvant endocrine therapy after R0 resection remains uncertain and requires further investigation.

## Data Availability

The raw data supporting the conclusions of this article will be made available by the authors, without undue reservation.
